# Uncovering the Effects and Molecular Mechanisms of Shaoyao Decoction Against Colorectal Cancer Using Network Pharmacology Analysis Coupled With Experimental Validation and Gut Microbiota Analysis

**DOI:** 10.1002/cam4.70813

**Published:** 2025-03-22

**Authors:** Yaojun Rong, Guiyu Zhang, Wenhao Ye, Linhua Qi, Xiaojiang Hao, Xiaolin Li, Wuhong Zhang, Yangfa Chao, Shaodong Gu

**Affiliations:** ^1^ Shenzhen Bao'an Traditional Chinese Medicine Hospital, Guangzhou University of Chinese Medicine Shenzhen Guangdong China; ^2^ The Seventh Clinical Medical College of Guangzhou University of Chinese Medicine Shenzhen Guangdong China

**Keywords:** 16S rRNA sequencing, colorectal cancer, gut microbiota, liquid chromatography–mass spectrometry (LC–MS), network pharmacology, Shaoyao decoction

## Abstract

**Background:**

Chronic gut inflammation and dysbiosis contribute significantly to colorectal cancer (CRC) development. Shaoyao decoction (SYD) is a well‐established Chinese medicine prescription. Besides ameliorating CRC via anti‐inflammatory effects, SYD modulates gut microbiota (GM) to improve inflammatory responses in ulcerative colitis (UC). However, whether and how SYD suppresses CRC by regulating GM remains largely unknown.

**Methods:**

SD rats were orally administered SYD for 7 days to obtain medicated serum. We utilized liquid chromatography–mass spectrometry (LC–MS) analysis, GeneCards, DisGeNET, and SwissTargetPrediction databases to analyze blank and SYD‐medicated rat serum, comparing the findings with those of SYD aqueous extract in previous studies to identify SYD circulating compounds/components with predictable target genes. Using network pharmacology, the potential active compounds and corresponding hub genes associated with modulating GM to suppress CRC were selected for molecular docking. In vivo experiments, a CRC transplantation tumor model was established in BALB/c mice using CT26 cells, with SYD gavage for 14 days. To investigate the mechanism of SYD‐regulated GM against CRC, HE and IHC staining, Western blotting, and 16S rRNA sequencing were employed.

**Results:**

LC–MS identified 26 SYD compounds with computationally predicted target genes. Network pharmacology prioritized 13 compounds targeting 8 inflammation/immunity‐related genes (IL‐17/TNF pathways), validated by molecular docking. In vivo experiments, SYD dose‐dependently suppressed tumor growth (*p* < 0.05, medium/high doses), as confirmed by HE staining and IHC analysis of Ki‐67. Notably, SYD potentially delayed CRC liver metastasis and alleviated hepatic injury in tumor‐bearing mice. Western blotting demonstrated SYD's inhibition of the IL‐17/TNF/NF‐κB axis, aligning with computational predictions. 16S rRNA sequencing revealed SYD‐enriched Akkermansia and GM structural shifts, mechanistically linking microbiota remodeling to anti‐tumor efficacy.

**Conclusions:**

SYD combats CRC via dual modulation of IL‐17/TNF/NF‐κB signaling and GM ecosystems (e.g., Akkermansia enrichment). This microbiota‐immune crosstalk positions SYD as a potential adjunct to conventional therapies, particularly for CRC patients with dysbiosis.

## Graphical abstract

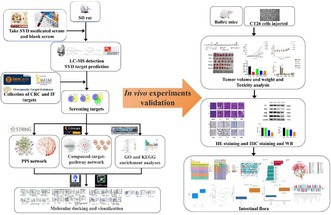

**Abbreviations**
5‐FU: 5‐Fluorouracil
ARE: anti‐oxidative response element
BCA: bicinchoninic acid
CA199: carbohydrate antigen 199
CASP3: Caspase‐3
CEA: carcinoembryonic antigen
CRC: colorectal cancer
CTAB: cetyltrimethylammonium bromide
CXCL8: C–X–C motif chemokine ligand 8
DAB: diaminobenzidine
DMEM: Dulbecco's modified Eagle's medium
EMT: epithelial–mesenchymal transition
EP: Eppendorf tube
EPC: edge percolated component
FBS: fetal bovine serum
GAPDH: glyceraldehyde‐3‐phosphate dehydrogenase
GM: gut microbiota
GO: Gene Ontology
GO‐BP: GO biological process
GO‐CC: GO cellular component
GO‐MF: GO molecular function
GPX4: glutathione peroxidase 4
HE staining: hematoxylin–eosin staining
HSYD: high‐dose Shaoyao decoction
IBD: inflammation bowel disease
IFN: interferon
IHC: immunohistochemistry
IL‐8: interleukin‐8
IκB: inhibitor of NF‐κB
IκK: inhibitor of kappa B kinase
JUN: Jun proto‐oncogene
KEGG: Kyoto Encyclopedia of Genes and Genomes
KO: KEGG Orthology
LC–MS: liquid chromatography–mass spectrometry
LDA: linear discriminant analysis
LEfSe: linear discriminant analysis effect size
LSYD: low‐dose Shaoyao decoction
MAPK: mitogen‐activated protein kinase
MCC: maximum clique centrality
MMP9: matrix metalloproteinase 9
MNC: maximal clique centrality
MSYD: medium‐dose Shaoyao decoction
NF‐κB: nuclear factor kappa‐B
NLRP6: NOD‐like receptor family pyrin domain containing 6
NOD: nucleotide oligomerization domain
Nrf2: nuclear factor erythroid 2‐related factor 2
OMIM: Online Mendelian Inheritance in Man
PCoA: principal co‐ordinate analysis
PD‐1: programmed death receptor 1
PLS‐DA: partial least‐squares discriminant analysis
PPI Networks: Protein–Protein Interaction Networks
PPRAG: peroxisome proliferator activated receptor gamma
PTGS2: prostaglandin‐endoperoxide synthase 2
RDA: redundancy analysis
RIP2: receptor‐interacting‐serine/threonine‐protein kinase 2
SCFAs: short‐chain fatty acids
STRING: search tool for the retrieval of interacting genes/proteins
SYD: Shaoyao decoction
TAM: tumor‐associated macrophage
TNF: tumor necrosis factor
TTD: Therapeutic Target Database
UHPLC: ultrahigh‐performance liquid chromatography

## 1 Introduction

The International Agency for Research on Cancer (IARC) forecasted that colorectal cancer (CRC) would top the global rankings of cancer in the next 50 years, as published in Nature [1]. The incidence rate and mortality of CRC remain high, ranking third and second, respectively, among all malignancies [2]. Precancerous polyps were the main source of CRC [3]. The serrated tumor pathway would lead to CRC, according to the most recent studies [4]. It is yet unknown how CRC works at the molecular level. Numerous risk factors, such as dietary and environmental factors, individual habits, family history, and genetic predispositions, were believed to contribute to the pathogenesis of CRC [5]. Following clinical surgery, formidable challenges arise regarding survival rates, drug resistance, and the toxicity of radiation and chemotherapy. Patients with early‐stage CRC exhibit a 90% 5‐year survival rate, whereas individuals with locally advanced disease have a 70% 5‐year survival rate, and those with metastatic colorectal cancer (mCRC) experience a 5‐year survival rate of only 15%. In fact, the majority of CRC patients in clinical practice have already been at intermediate to advanced stages of the disease when they received a diagnosis [6]. Therefore, it is crucial and urgent to discover novel treatments as well as safer and more efficient clinical trial medications.

Reports suggest that chronic intestinal inflammation and dysbiosis exert a crucial role in the development of CRC. Persistent chronic inflammation leads to CRC by facilitating the accumulation of DNA damage and mutations, activating the immune system for abnormal cell proliferation and carcinogenesis, inducing aberrant DNA methylation and histone modifications, and triggering dysregulated activation of key signaling pathways, such as nuclear factor kappa‐B (NF‐κB) and JAK/STAT, to promote cell proliferation and inhibit apoptosis [7]. Imbalanced gut microbiota (GM) can precipitate mucosal barrier disruption, culminating in chronic inflammation of the tissue and the secretion of inflammatory and pro‐carcinogenic mediators, thereby increasing the risk of developing CRC [8]. A strong relationship between the GM and the development of CRC has been demonstrated by research in recent years, which has made it possible to seek a new approach to treating CRC. Through the production of toxic compounds and modifications to the host's physiological processes, the ecological imbalance of the GM could contribute to the onset and progression of CRC [9]. According to studies, controlling the GM and its metabolites enhanced the therapeutic impact while mitigating the negative effects of 5‐fluorouracil (5‐FU), a commonly used drug for gastrointestinal tumors [10]. Inflammation and apoptosis of the colonic mucosa, which may be related to the regulation of the NF‐κB, Nrf2‐ARE, and MAPK/P38 pathways, could be reduced by the GM and its metabolites [11]. GM is recognized as being a promising avenue for the treatment of CRC.

Traditional Chinese medicine provides considerable therapeutic advantages in modulating the GM, based on its fundamental philosophy, the distinctive approach to syndrome differentiation and treatment, and its synergistic application with other healing techniques [12]. Shaoyao decoction (SYD) is a widely utilized Chinese medicine prescription, which has the function of removing heat, drying moisture, and maintaining the harmony of qi and blood based on traditional Chinese medicine theories. Baishao (BS), Danggui (DG), Huanglian (HL), Binlang (BL), Muxiang (MX), Zhigancao (ZGC), Dahuang (DH), Huangqin (HQ), and Guangui (GG) are among the nine herbs that make up the formula. The detailed information in botany with daily recommended dosage for adults can be found in (Table 1). SYD is frequently used to treat moist heat dysentery in clinical settings and also inhibits the growth of CRC. According to the research, SYD mitigates acute and chronic colitis and reduces the risk of colitis‐associated CRC by modulating the Keap1–Nrf2–ARE pathway, thereby inhibiting inflammation and oxidative stress [13]. Specific properties of SYD, through their anti‐inflammatory actions and modulation of the GM, have significantly enhanced the prognosis for rats afflicted with moist heat‐induced diarrhea. The key to SYD promoting the rebalance of GM is attributed to the inclusion of *Rheum palmatum* L. in its formulation, which decreases the number of harmful bacteria and encourages the growth of probiotics [14]. In conclusion, The well‐established traditional Chinese medicine formula SYD has shown positive effects in anti‐inflammation, immune regulation, and maintaining GM balance in the treatment of UC and CRC.

**TABLE 1:** Composition and dosage of SYD.

Herb of Chinese medicine	Botanical name*	Daily recommended dosage for adults	Batch number of herb pieces	The origin of herbs
Baishao (BS)	*Paeonia lactiflora* Pall.	30 g	No. 2102181	Anhui, China
Danggui (DG)	*Angelica sinensis* (Oliv.) Diels.	15 g	No. 210620	Gansu, China
Huanglian (HL)	*Coptis chinensis* Franch.	15 g	No. 201220	Chongqing, China
Binlang (BL)	*Areca catechu* L.	6 g	No. 20210301	Guangxi, China
Muxiang (MX)	*Aucklandia costus* Falc.	6 g	CP‐030‐201201	Yunnan, China
Zhigancao (ZGC)	*Glycyrrhiza uralensis* Fisch.	6 g	No. 210601	Inner Mongolia, China
Dahuang (DH)	*Rheum palmatum* L.	9 g	No. 20210501	Chongqing, China
Huangqin (HQ)	*Scutellaria baicalensis* Georgi.	15 g	No. 210401	Shanxi, China
Guangui (GG)	*Cinnamomum cassia* (L.) D. Don.	7.5 g	No. 2106034	Guangxi, China

* The botanical name has been confirmed by https://www.plantplus.cn/cn.

However, there is currently a lack of direct evidence regarding the pharmacological mechanism of SYD through the interaction of GM, immune pathway, and CRC. Despite the promising effects of SYD in treating UC and CRC, several critical gaps remain in understanding its pharmacological mechanisms. The precise mechanisms by which SYD modulates the GM in individuals with CRC are not fully understood, nor is it clear whether these changes directly contribute to the inhibition of CRC. In addition, the optimal dosage of SYD for achieving these regulatory effects remains to be determined, as different dosages may produce distinct outcomes. Could SYD be a new therapeutic option for CRC patients with concomitant GM dysbiosis? These existing gaps in the current research field provide us with some entry points for our study.

Considering SYD as a prescription composed of various herbs and components, presumably having multiple active ingredients that enter the body and participate in metabolism to exert therapeutic effects, in order to analyze the potential active components of SYD more comprehensively and efficiently, LC–MS analysis [15] was utilized for SYD aqueous extract (completed in previous studies) [16], SYD‐containing rat serum, and blank rat serum to analyze the composition, identify, and quantify various metabolites for the subsequent screening of network pharmacology.

Network pharmacology, an efficient and holistic scientific research method, has been extensively utilized in the study of ethno drugs or herbal medicine [17, 18]. Important target genes were selected through Venn diagram and protein–protein interaction (PPI). Gene functions were annotated based on databases such as Kyoto Encyclopedia of Genes and Genomes (KEGG) and Gene Ontology (GO). GO analysis helped identify the biological processes (BP), molecular functions (MF) and cellular components (CC) that might be influenced by the active components of SYD. By comparing differentially expressed genes between the experimental and control groups, we discovered the gene functions and biological pathways that SYD's active components might regulate GM to affect CRC at the molecular level. KEGG analysis was employed to determine the signaling pathways that might be affected by the active components of SYD. By analyzing the gene targets related to CRC, the key signaling pathways through which SYD's active components might exert their effects to regulate GM were identified. Through the above analyses, network pharmacology helped explore the molecular mechanism by which SYD, capitalizing on its pharmacological characteristics of multi‐component, multi‐target, and multi‐channel action, modulates the GM to combat CRC.

In the present study, we screened SYD circulating components with predictable target genes by using LC–MS, predicted the common target genes and functioning pathways of SYD in the GM‐regulated treatment of CRC, and identified SYD potential active components and corresponding hub genes through network pharmacology and molecular docking. According to the findings of network pharmacology, we conducted in vivo experiments to assess the impact of SYD on tumor‐bearing mice with CT26 cell transplants based on various parameters, including the volume and mass of tumors, the levels of necrosis and apoptosis within tumor cells, liver damage, and intestinal inflammatory metabolism. Finally, high‐throughput sequencing (16S rRNA sequencing) was used to track changes in GM in the mice's feces. A stronger foundation for further investigation of the molecular mechanism of SYD controlling GM against CRC was provided by the analysis of the diversity and differences of GM in tumor‐bearing mice.

## 2 Materials and Methods

### 2.1 Animals and Cells

Animals utilized in this research were 50 female BALB/c mice (#L2110211), aged 3–4 weeks and weighing 10–15 g, along with 10 male SD rats aged 5–6 weeks and weighing 200 g (#L2112011). These animals were procured from Guangzhou Dean Gene Technology Co. Ltd., adhering to the ethical standards of Shenzhen Top Biotech Co. Ltd. Institutional Animal Care and Use Committee, IACUC (No. TOP‐IACUC‐2021‐0125 for SD rats, No. TOP‐IACUC‐2021‐0126 for BALB/c mice). The animal housing facilities provided a regulated environment with a temperature of 20°C ± 2°C, a 12‐h light–dark cycle, and ad libitum access to food and water.

Pricella (Wuhan, China) supplied Dulbecco's modified Eagle's medium (DMEM) containing 10% fetal bovine serum (FBS), 1% penicillin, and 1% streptomycin (#PM150210B), used to cultivate the CT26 murine colon cancer cells (provided by the National Collection of Authenticated Cell Cultures in Shanghai, China) at 37°C and 5% CO_2_.

### 2.2 Main Antibody and Reagents in the Experiment

All the main reagents and antibodies involved in the research refer to Data S1.

### 2.3 Preparation of SYD Water Extract

The raw herb pieces for SYD were obtained from the Pharmacy of Shenzhen Bao'an District Traditional Chinese Medicine Hospital. The daily dosage for decoction [13] and detailed batch numbers of the herb pieces are shown in (Table 1). According to the proven drug preparation method from the previous study [16], The raw herb pieces were then mixed according to the prescribed proportion of each herb. Then, the mixture of 109.5 g SYD was soaked in distilled water for 30 min and decocted for 1 h at 100°C by adding 10× (1st round) and 6× (2nd round) volume of distilled water. After extractions, combined with the decoction from both times, the SYD filtrate was centrifuged twice at 5000 rpm for 10 min (Refrigerated benchtop centrifuge, Sigma Laborzentrifugen #SIGMA 4‐16KS, Osterode am Harz, Germany) to remove tiny herbal residues and concentrated using a rotary evaporator (Yarong #RE‐3000, Shanghai, China) to achieve a drug concentration of 2 g/mL (calculated with raw herbs). Finally, the decoction was then aliquoted into 4‐mL tubes and frozen at −20°C for later use.

### 2.4 Preparation of SYD‐Containing Rat Serum

Referring to the established protocol for medicated serum preparation as reported in previous research [19], after 7 days of adaptive feeding, SD rats were evenly split into Control and SYD groups based on their body weight. Every rat in the SYD group received 1.971 mL (9.855 g/kg) of SYD water extract by gavage once daily for 7 days, while the Control group received 1.971 mL of physiological saline via gavage once daily. On the seventh day, 1–2 h after giving the rats SYD by gavage, blood was collected from their abdominal aortas. SD rats fasted for 12 h prior to the final oral administration of SYD. All abdominal aortic blood samples from SD rats were centrifuged twice at 3500 rpm for 15 min each (Refrigerated microcentrifuge, Sigma Laborzentrifugen #SIGMA 1‐14KS, Osterode am Harz, Germany) after being left at room temperature for 1 h. Finally, the serum was gathered, passed through a 0.22‐μm filter (Millipore #SLGP033RB, Billerica, MA, USA), and then stored at −80°C.

### 2.5 LC–MS Analysis

LC–MS analysis was conducted [20] to scrutinize the blank rat serum (from SD rats blood serum of control group), SYD‐containing rat serum (from SD rats blood serum of SYD group) and SYD aqueous extract. Each Eppendorf (EP) tube was aliquoted with 100 μL of SYD aqueous extract, SYD‐containing rat serum, and blank rat serum. The contents were then thoroughly resuspended with pre‐cooled 80% methanol by well‐vortexing. The samples were incubated for 5 min on ice before being centrifuged at 15,000 g for 20 min at 4°C. LC–MS grade water (Merck #1.15333, Rahway, NJ, USA) was used to dilute some of the supernatant to a final methanol concentration of 53%. The samples were then put into brand‐new EP tubes and centrifuged for 20 min at 15,000 g and 4°C. The supernatant was added to the LC–MS/MS apparatus (Thermo Fisher Scientific Q Exactive #0726060, Waltham, MA, USA) for analysis [21]. Compound finder 3.1 (CD3.1, ThermoFisher) was used to handle the UHPLC–MS/MS raw data file in order to perform peak alignment, peak picking, and quantification for each metabolite. For statistical analysis, R (version R‐3.4.3), Python (version Python 2.7.6), and CentOS (version CentOS 6.6) were utilized. The area normalization approach was implemented for normal transformation when the data do not follow a normal distribution.

### 2.6 Network Pharmacology

The chemical components of SYD‐containing rat serum, blank rat serum, and SYD water extract were ascertained using LC–MS analysis. The medicinal components potentially linked to SYD were initially screened by analyzing the intersection of SYD‐containing rat serum and blank rat serum. Subsequently, these components were further cross‐referenced with the water extract of SYD to identify the specific components that enter the bloodstream. Further screening of SYD components in rat serum was conducted [17] using the SwissADME database, with criteria including a GI and Druglikeness score greater than three YES. The screened components were fed into the SwissTargetCondition database to determine target genes, which subsequently were entered into the PubChem database to search for 2D structural formulae. The Gene Cards, OMIM, and TTD databases were used to identify target genes associated with CRC and GM. To create a Venn diagram, the target genes of SYD, CRC, and GM were intersected. Utilizing Cytoscape 3.8.0, component target network diagrams, PPI diagrams, and drug‐disease‐microbiota component target communication network diagrams were created; The microbiome website was utilized to generate KEGG bubble charts and GO bar charts. The intersected target genes identified were then input into the Metascape website to perform GO and KEGG enrichment analysis. Based on various algorithms, the top 10 targets were screened through the Cytohubba plugin for the purpose of discovering hub genes and their associated chemicals. Autodock Tools 1.5.6 was applied to pretreat proteins and small molecular compounds such as water removal and hydrogenation, followed by performing Macromolecular docking and using Pymol 2.6.0 to visualize the results.

### 2.7 Homozygous Mouse Model and Animal Grouping

Adaptively feeding the purchased female BALB/c mice for 7 days. CT26 cells from the logarithmic growth stage (fusion rate 60%–80%, 4th passage) were transplanted into the right armpits of mice, following the protocol established by [22, 23] for constructing tumor‐bearing mouse models. The tumor cells at 1 × 10^5^ volume were transplanted per mouse, completed within half an hour. A week after the tumor transplantation, palpable small tumors were detected in the mice's armpits. Subsequently, these tumor‐bearing mice were randomly separated into five groups, each consisting of 10 mice: A CT26 transplantation‐induced tumor model group (Model group, only gavaged with 0.3mL physiological saline per mouse); a CT26 transplantation‐induced tumor model treated by clinical dose SYD group (namely middle‐dose SYD group, MSYD group, 19.2 g/kg/day via gavage); a CT26 transplantation‐induced tumor model treated by high‐dose SYD group (HSYD group, 38.4 g/kg/day via gavage); a CT26 transplantation‐induced tumor model treated by low‐dose SYD group (LSYD group, 9.6 g/kg/day via gavage); and a CT26 transplantation‐induced tumor model treated by 5‐FU clinical dose group (5‐FU group, as the positive control group, 104 mg/kg/2 day by intraperitoneal injection). The treatment cycle lasted for 2 weeks. All five groups of tumor‐bearing mice were given regular feed and free sterile water for drinking.

### 2.8 Collection and Preparation of Tumors, Organs, and Feces Samples

The tumor dimensions, including maximum length (marked *d*_max_) and transverse diameter (marked *d*_min_), were assessed every 48 h, with tumor volume (marked *V*) subsequently calculated using the established formula: $V=\pi \times {\left(\frac{d_{\max}}{2}\right)}^{2}\times d_{\min}$. During this 2‐week period, the feces of mice were collected to detect the changes of GM. At the end of the experiment, all five groups of mice were humanely euthanized via cervical dislocation, followed by the tumors and main organs (liver, heart, spleen, lung and bilateral kidneys) dissected and weighed. Portions of the tumor tissues were excised and fixed in 4% paraformaldehyde for hematoxylin–eosin (HE) staining and immunohistochemistry (IHC) staining. The rest were frozen in liquid nitrogen, then transferred to the refrigerator at −80°C for Western blotting detection.

### 2.9 HE and IHC Staining

Drawing on the methodologies used in similar studies [24], the tumor tissues were sectioned (Manual microtome, Leica #RM2016, Wetzlar, Germany) after being fixed in 4% paraformaldehyde. Slices were sequentially placed into water containing 75% alcohol by volume of xylene, anhydrous ethanol for dewaxing (Automatic tissue dehydrator, DIAPATH #Donatello SDSDN9000, Milan, Italy). Thereafter, HE staining, dehydration (Automatic tissue dehydrator, DIAPATH #SDSDN9000, Italy), sealing, microscopic inspection (Upright Microscopes, Nikon #ECLIPSE E100, Tokyo, Japan), picture collecting (Camera Control Unit, Nikon #DS‐U3, Tokyo, Japan), and image analysis were performed. IHC investigations were carried out after the tissue sections had been dewaxed and brought to water (Automatic tissue dehydrator, DIAPATH #SDSDN9000, Italy). After repairing antigens and blocking endogenous peroxidase activity (See‐Saw rocking shaker #DS‐2S100, Servicebio, Wuhan, China), mouse serum was blocked. The primary antibody Ki67 was then applied at a 1:1000 dilution for overnight incubation. Subsequently, the sections were added with a secondary antibody, followed by diaminobenzidine (DAB) staining to visualize the antigen–antibody complexes. The nuclei were then re‐stained, and the sections were subsequently dehydrated and sealed to complete the preparation for microscopic investigation (Upright Microscopes, Nikon #E100, Japan) and analysis of the picture data (Camera Control Unit, Nikon #DS‐U3, Japan).

### 2.10 Western Blotting

Still referring to the method of [24], tumor tissues belonging to the same group of mice were mixed in equal volumes, along with lysate, phosphatase inhibitors, and protease inhibitors. The mixture was ground (Cryogenic grinding mill, Luka #LUKYM‐II, Guangzhou, China), allowed to stand for 30 min, and then centrifuged twice at 12,000 rpm for 30 min each. The supernatant was then collected. 6 μg/μl of the bicinchoninic acid (BCA) quantitative unified concentration was collected. Subsequently, protein samples were separated by gel electrophoresis (Vertical electrophoresis apparatus, Bio‐Rad #1658029, Hercules, CA, USA) and transferred to a membrane (Trans‐Blot Turbo Transfer System, Bio‐Rad #1704150, Hercules, CA, USA). After blocking, the membrane was incubated with primary antibodies (p65, p‐p65, IκB, p‐IκB) at a concentration of 1:1000 and GAPDH (internal reference) at 1:2000 overnight. Following incubation with a secondary rabbit‐specific antibody at 1:2000, the bands were developed and analyzed (Chemiluminescence Imaging System, Tanon #5200multi, Shanghai, China).

### 2.11 16S rRNA Sequencing

Before the end of the experiment, the feces of all groups of mice were collected for GM testing according to the supplier's requirements [25]. Fecal genomic DNA was extracted with CTAB, and then the purity and concentration of DNA were detected by 1% agarose gel electrophoresis. An appropriate amount of feces sample was placed in a centrifuge tube and diluted to 1 ng/μl with sterile water. 341F (5′‐CCTAYGGRBGCASCAG‐3′) and 806R (5′‐GGACTACNNGGGTATCTAAT‐3′), v3 + v4 can PCR amplification of the variable region was performed. Sequencing libraries were generated using the TRUSEQ DNA PCR Free Sample Preparation Kit (Illumina, USA) according to the manufacturer's recommendations, and index codes were added. Library quality was evaluated on a quantum bit @ 2.0 fluorescence meter (Thermo Scientific). Finally, libraries were sequenced on the Illumina NovaSeq platform (High‐throughput sequencer, Illumina #PE250, San Diego, CA, USA) and 250 bp paired‐end reads were generated. PLS‐DA (partial least‐squares discriminant analysis) was also introduced as a supervised model to reveal inter‐group microbiota variation using the “plsda” function [26] in the R package “hybridomics” Redundancy analysis (RDA) was used to reveal associations between microbial communities and environmental factors, based on the relative abundance of microbial species at the level of different taxa, using the R package “vegan” (Jari Oksanen, F. Guillaume Blanchet, Michael Friendly et al. 2020). Co‐occurrence analysis was performed by calculating Spearman's rank correlation between dominant taxa and using network plots to show associations between taxa. In addition, potential KEGG direct homology (KO) functional profiles of microbial communities were predicted using PICRUSt [27]. Parameters used in the analysis were set to default values unless specified above.

### 2.12 Statistical Analysis

All measurement data were divided into normal measurement (“expressed by mean plus minus standard deviation $\left(\bar{x}\pm s\right)$”), non‐normal measurement (“expressed by average rank sum ($\bar{R}$) and Quartile (*P25, P50, P75*)”), counting “expressed by frequency (*ƒ*), constituent ratio or percentage (*P*) and average rank sum ($\bar{R}$),” and large sample counting data “expressed by frequency (*ƒ*), constituent ratio or percentage (*P*) and average Radit value ($\bar{R}$).” All data were analyzed using *IBS SPSS Statistics 23* software. Significant differences between groups were considered when *p* < 0.05 (*p < 0.05, **p < 0.01, ***p < 0.001).

## 3 Results

### 3.1 LC–MS Analysis

**FIGURE 1:**
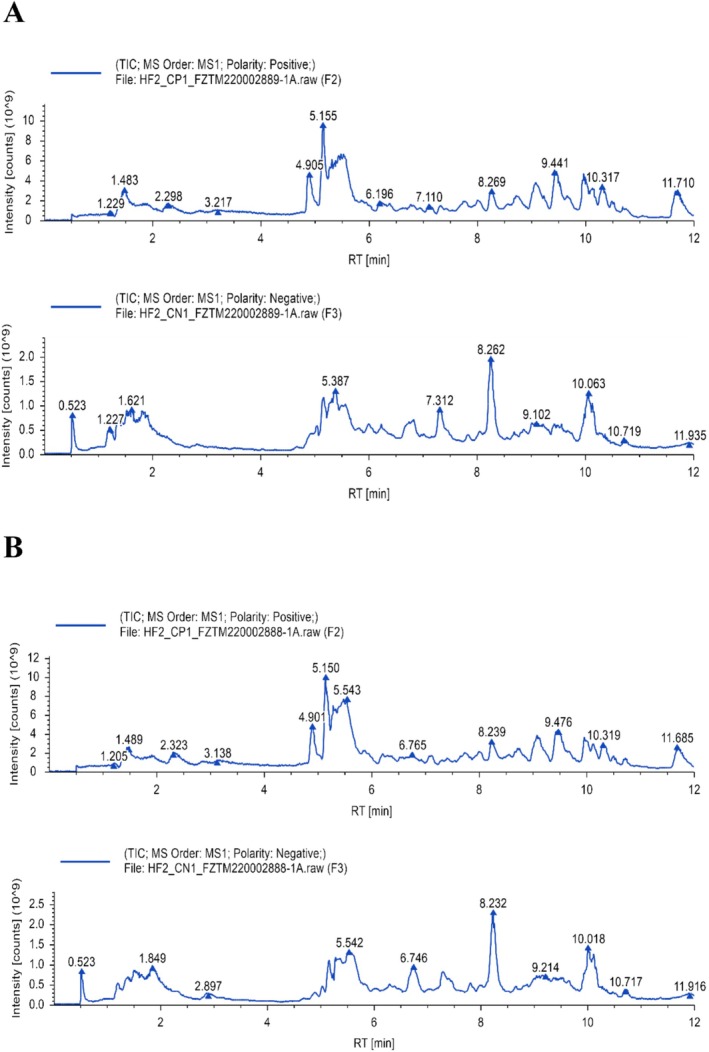
The compositions of SYD‐containing rat serum and blank rat serum were determined by LC‐ MS. (A) Total ion diagram of SYD‐containing rat serum in both positive and negative modes. (B) Total ion diagram of blank rat serum in both positive and negative modes.

**FIGURE 2:**
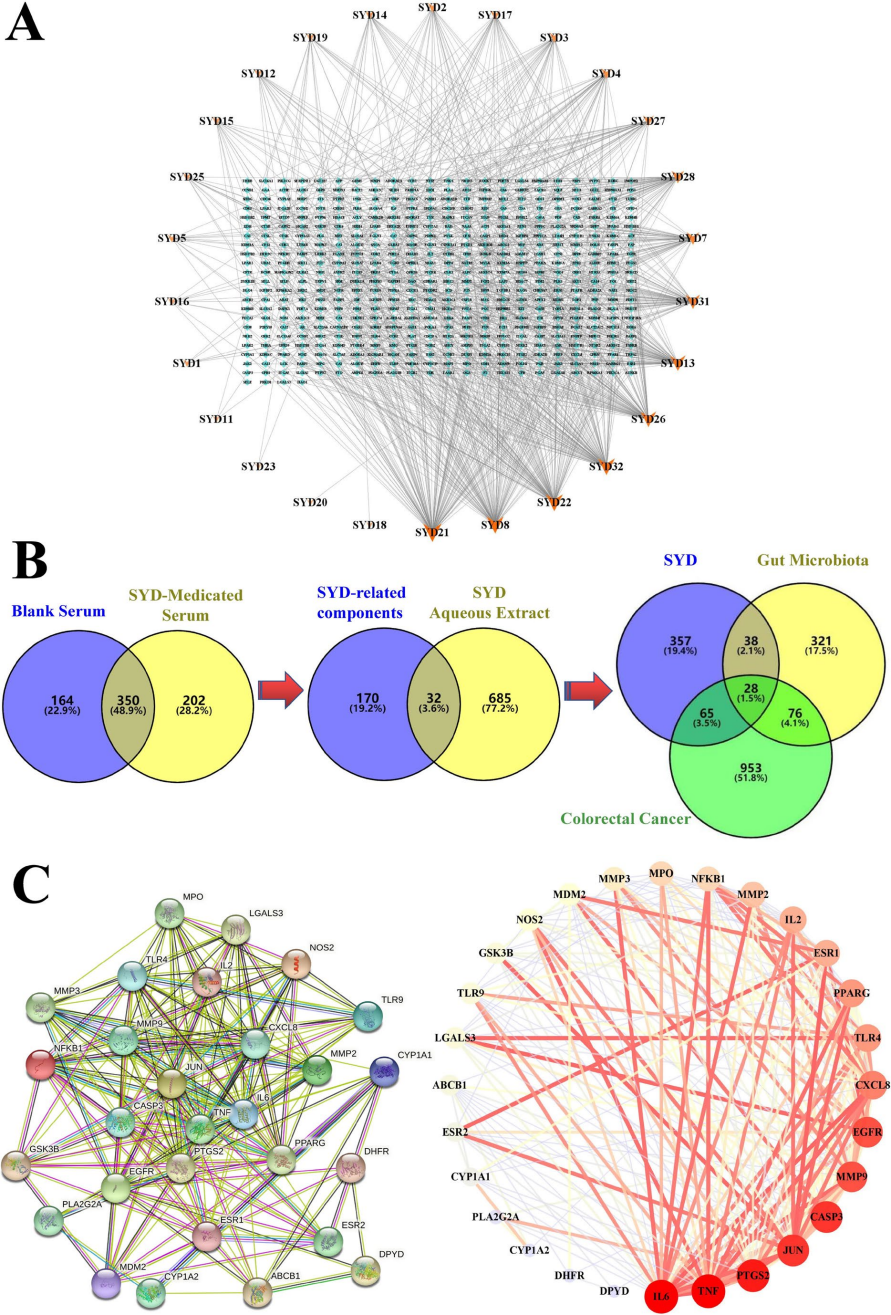
Network pharmacological results. (A) The compound‐target network. (B) Venn diagrams illustrating 202 SYD‐related compounds identified, further selecting 32 circulating compounds of SYD and comparing the numbers of shared target genes among SYD, GM, and CRC. (C) PPI network of the drug–microbiota–disease targets.

Using LC–MS analysis, 552 chemicals in total were found in SYD‐containing rat serum and 514 compounds in blank rat serum, as shown in Figure 1A,B. There were 717 different substances that were taken out of SYD water in total for the preliminary investigation [16]. A total of 32 compounds were chosen after choosing the intersection, and 26 of these could predict target genes (see Table 2). Four hundred and eighty‐eight target genes were chosen after screening, as shown in Figure 2A. A total of 1122 target genes for CRC and 463 target genes for GM were obtained from the respective GeneCards, OMIM, and TTD databases. Twenty‐eight target genes were screened following intersection, with a Venn diagram displayed in Figure 2B.

**TABLE 2:** Predicted target genes of 26 circulating compounds.

Name	Formula	Molecular weight	RT [min]	*m*/*z*
Citric acid	C_6_H_8_O_7_	192.02659	1.666	191.01932
DL‐α‐methoxyphenylacetic acid	C_9_H_10_O_3_	166.06256	5.285	165.05528
Trans‐cinnamic acid	C_9_H_8_O_2_	148.05211	5.619	147.04483
Syringic acid	C_9_H_10_O_5_	198.05597	5.383	197.04869
4‐Hydroxybenzylalcohol	C_7_H_8_O_2_	124.05214	5.501	123.04486
Daidzein	C_15_H_10_O_4_	254.05746	8.28	253.05019
Genistein	C_15_H_10_O_5_	270.05253	7.872	269.04526
2‐(5‐mercapto‐4‐methyl‐4H‐1,2,4‐triazol‐3‐yl)acetonitrile	C_5_H_6_N_4_S	154.02955	5.286	153.02228
Mevalonic acid	C_6_H_12_O_4_	148.07321	4.784	147.06593
Propylparaben	C_10_H_12_O_3_	198.0888	5.854	197.08165
NG,NG‐dimethyl‐L‐arginine	C_8_H_20_C_l2_N_4_O_2_	274.09523	5.599	273.08795
PC (16:0/16:1)	C_40_H_78_NO_8_P	731.54601	9.597	732.55328
N‐acetylneuraminic acid	C_11_H_19_NO_9_	309.10567	1.901	308.09839
3‐(2‐naphthyl)‐D‐alanine	C_13_H_13_NO_2_	215.09447	4.867	216.10175
Choline	C_5_H_13_NO	103.09952	1.412	104.1068
Coumarin	C_9_H_6_O_2_	146.03661	5.781	147.04388
Thymine	C_5_H_6_N_2_O_2_	126.04265	4.07	127.04993
Wogonin	C_16_H_12_O_5_	284.06801	6.516	285.07529
Epitestosterone	C_19_H_28_O_2_	288.20842	7.53	289.2157
DL‐arginine	C_6_H_14_N_4_O_2_	174.11142	1.42	175.1187
2‐(2‐acetyl‐3,5‐dihydroxyphenyl)acetic acid	C_10_H_10_O_5_	192.04207	5.827	193.04935
Leucylproline	C_11_H_20_N_2_O_3_	228.14719	4.791	229.15446
1‐Phenyl‐3‐methyl‐5‐pyrazolone	C_10_H_10_N_2_O	174.07924	4.749	175.08652
1‐Methyladenosine	C_11_H_15_N_5_O_4_	281.11171	1.862	282.11899
Valylproline	C_10_H_18_N_2_O_3_	214.13175	4.783	215.13902
3‐[4‐methyl‐1‐(2‐methylpropanoyl)‐3‐oxocyclohexyl]butanoic acid	C_15_H_24_O_4_	290.1513	6.633	291.15845

### 3.2 Network Pharmacology Analysis

**FIGURE 3:**
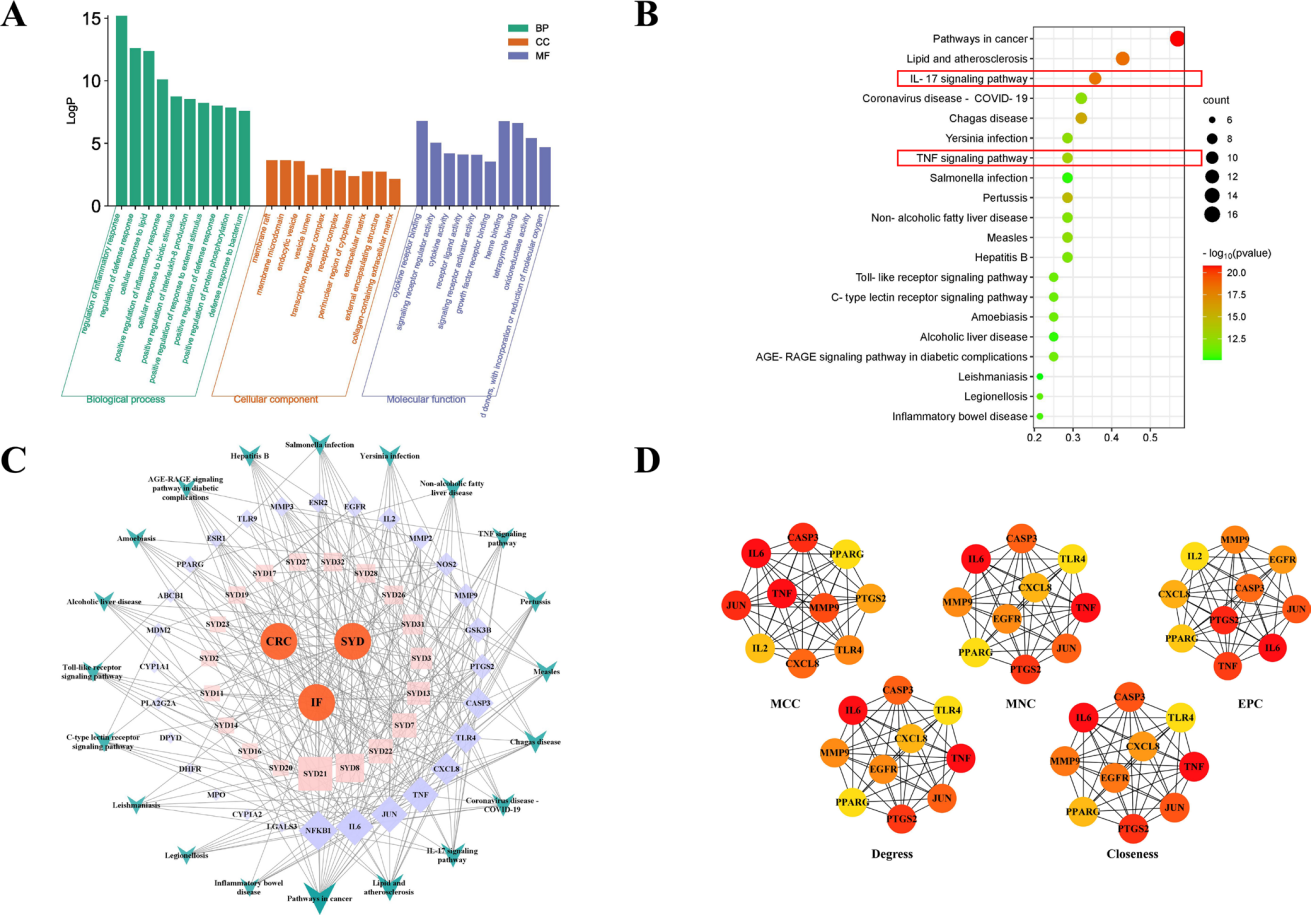
GO and KEGG enrichment analysis, the disease–microbiota–drug–compound–target–pathway network, and Hub genes. (A) Biological process, cellular component, and molecular function results of GO enrichment analysis. (B) Results of KEGG enrichment analysis. (C) The disease–microbiota–drug–compound–target–pathway network. (D) Hub genes calculated using MCC, MNC, EPC, Degree, and Closeness metrics. The color gradient of the circles, ranging from red to orange to yellow, reflects the varying degrees from high to low.

**FIGURE 4:**
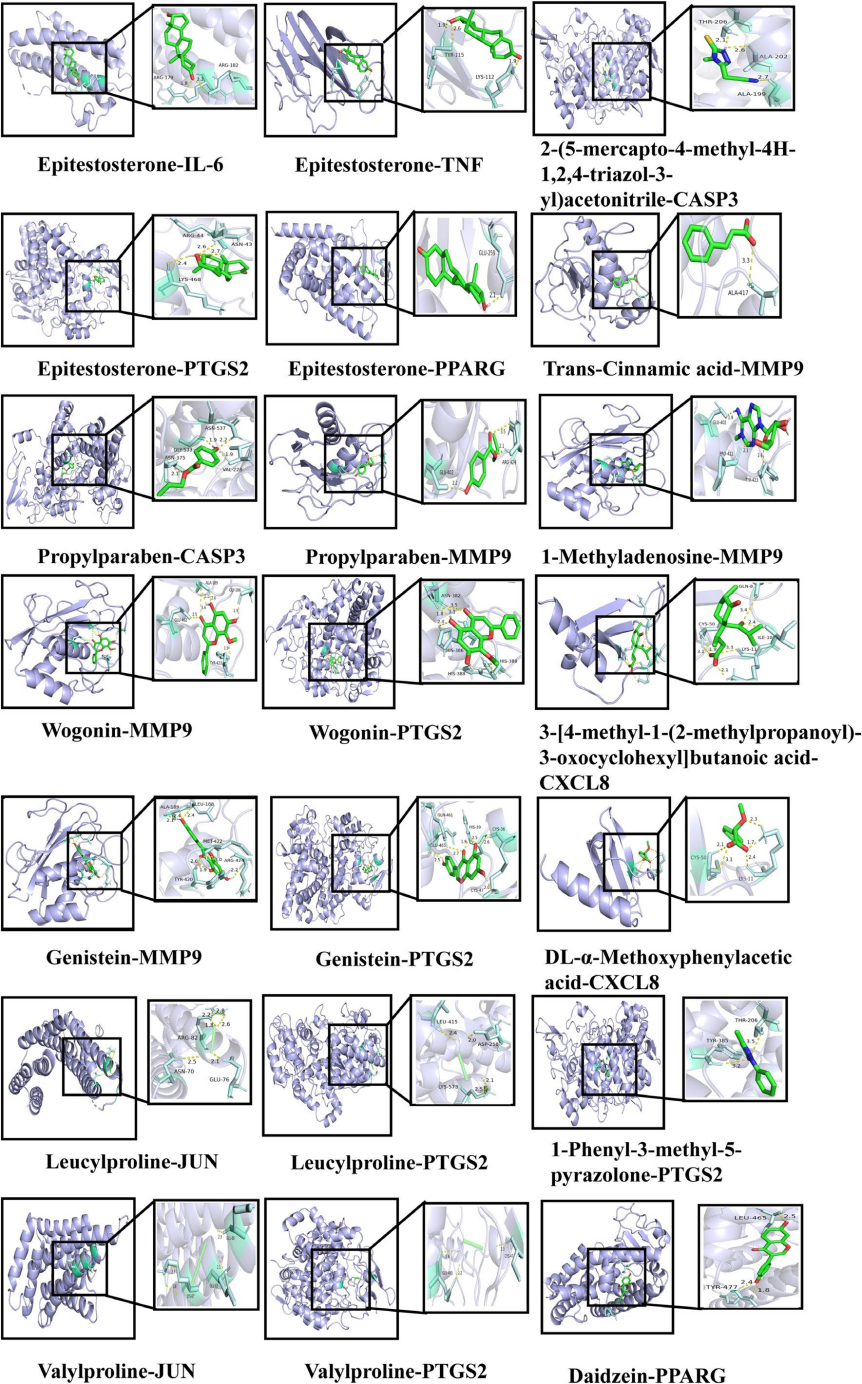
Molecular docking results.

Protein–protein interaction research involving 28 intersecting targets was conducted using the STRING database. Rustic analysis revealed a total of 28 nodes and 207 edges, with an average node degree of 14.8. The results were visually represented in Figure 2C. Enrichment analysis findings were depicted in Figure 3A,B. The top 20 pathways, such as IL‐17 signaling, TNF signaling, and the cancer pathway, were primarily identified through KEGG pathway enrichment. For the creation of a network diagram illustrating the medication‐component‐disease‐microbiota‐target‐pathway (Figure 3C), the top 20 signaling pathways from the KEGG pathway enrichment analysis were selected along with key action targets. GO‐BP functional analysis prioritized biological processes such as the regulation of inflammatory response and defense response, and positive regulation of interleukin‐8 production among other top 10 biological processes. GO‐CC functional analysis identified the top 10 cellular compounds such as membrane raft, membrane microdomain, and transcription regulator complex. The GO‐MF functional analysis revealed cytokine receptor binding, signaling receptor regulator activity, and cytokine activity among the top 10 molecular functions. Using Cytoscape 3.8.0 software for visualization and the cytoHubba plugin, we applied five algorithms—MCC, MNC, EPC, Degree, and Closeness—to screen the top 10 Hub genes. By merging the intersections, we identified the final 8 Hub genes, including IL6, TNF, JUN, CASP3, MMP9, CXCL8, PTGS2, and PPARG (see Figure 3D), which corresponded to 13 compounds such as Epitestosterone, 2‐(5‐mercapto‐4‐methyl‐4 H‐1,2,4‐triazol‐3‐yl)acetonitrile, propylparaben, leucylproline, valylproline, trans‐cinnamic acid, genistein, wogonin, 1‐methyladenosine, 3‐[4‐methyl‐1‐(2‐methylpropanoyl)‐3‐oxocyclohexyl]butanoic acid, DL‐α‐methoxyphenylacetic acid, 1‐phenyl‐3‐methyl‐5‐pyrazolone, and daidzein. The molecular docking analysis of the Hub genes and compounds was performed 21 times, and the results are shown in (Table 3) and visualized in (Figure 4). The docking studies indicated that complexes with binding free energy below −4 kcal/mol exhibited favorable interactions [28, 29].

**TABLE 3:** The molecular docking of the Hub genes and compounds.

Compound name	Target	PDB ID	Affinity (kcal·mol^−1^)
Epitestosterone	IL6	1alu	−6.7
Epitestosterone	TNF	1A8M	−7.17
Epitestosterone	PTGS2	5F1A	−8.25
Epitestosterone	PPARG	1ZGY	−7.68
2‐(5‐mercapto‐4‐methyl‐4H‐1,2,4‐triazol‐3‐yl)acetonitrile	CASP3	5IKR	−4.7
Propylparaben	CASP3	5IKR	−4.59
Propylparaben	MMP9	1GKC	−5.49
Leucylproline	JUN	6Y3V	−5.74
Leucylproline	PTGS2	5F1A	−4.84
Valylproline	JUN	6Y3V	−5.11
Valylproline	PTGS2	5F1A	−5.84
Trans‐cinnamic acid	MMP9	1GKC	−5.35
Genistein	MMP9	1GKC	−8
Genistein	PTGS2	5F1A	−5.68
Wogonin	MMP9	1GKC	−7.51
Wogonin	PTGS2	5F1A	−5.64
1‐methyladenosine	MMP9	1GKC	−4.66
3‐[4‐methyl‐1‐(2‐methylpropanoyl)‐3‐oxocyclohexyl]butanoic acid	CXCL8	3IL8	−6.97
DL‐α‐methoxyphenylacetic acid	CXCL8	3IL8	−5.81
1‐phenyl‐3‐methyl‐5‐pyrazolone	PTGS2	5F1A	−5.99
Daidzein	PPARG	1ZGY	−5.58

### 3.3 SYD Significantly Inhibited the Growth of Tumors in Tumor‐Bearing Mice and Reduced the Toxicity to the Liver

**FIGURE 5:**
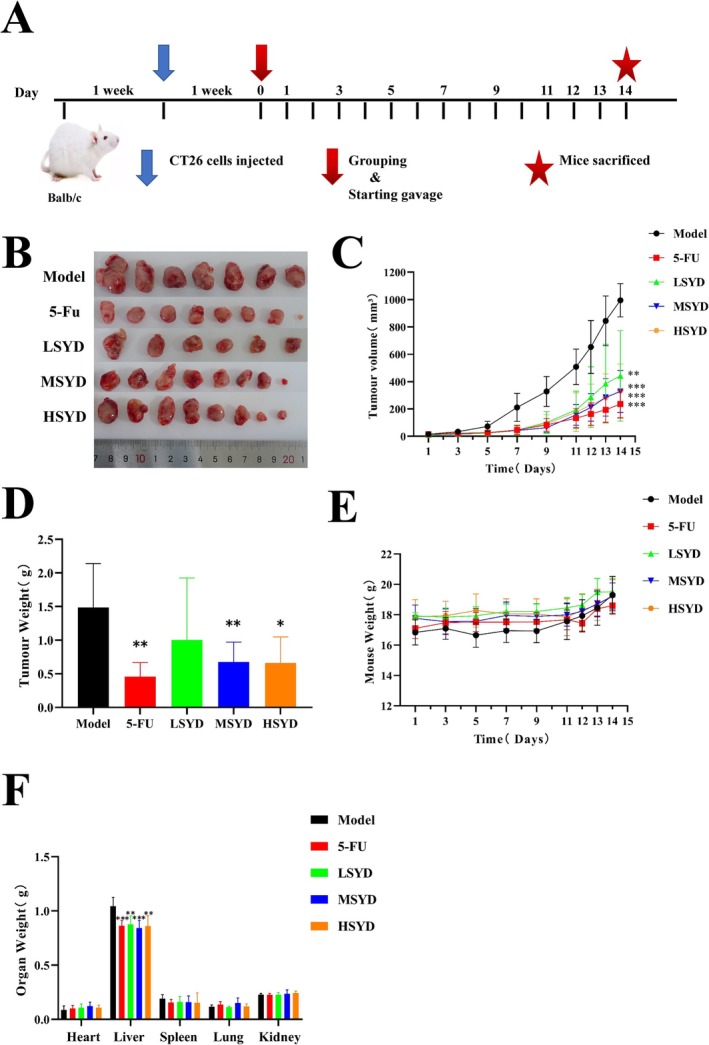
Experimental verification. (A) Animal experiment process. (B) Images of resected tumors from mice. (C, D) The volume and weight of tumors. (E) Changes in the body weight of mice during the experiment. (F) The weight of the heart, liver, spleen, lung, and bilateral kidneys *p < 0.05, **p < 0.01, ***p < 0.001 vs. control group.

In order to access the therapeutic efficacy of SYD on CRC, tumors were induced in BALB/c mice by subcutaneous injection of CT26 cells. Figure 5A presents the timeline of the animal experiment. Compared with the Model group, mice in the 5‐FU, LSYD, MSYD, and HSYD groups exhibited significantly reduced tumor sizes, as demonstrated in Figure 5B. Figure 5C illustrates the tumor volume changes throughout the gavage treatment phase. Compared to the Model group, the 5‐FU group exhibited the slowest rate of tumor growth, and the tumor volumes in the 5‐FU, LSYD, MSYD, and HSYD groups were significantly reduced (*p* < 0.001). Figure 5D presents the body weight fluctuation of the mice during gavage, while Figure 5E represents the post‐dissection tumor weights. The 5‐FU and MSYD groups had significantly lower tumor weights than the Model group (*p* < 0.01), and the HSYD group also showed a lower tumor weight (*p* < 0.05). Figure 5F displays the organ weights post‐dissection. The Model group exhibited a high metastatic potential, hepatomegaly, and significant liver injury (*p* < 0.01). SYD significantly retarded tumor growth and mitigated liver damage in tumor‐bearing mice.

### 3.4 SYD Promotes Tumor Cell Necrosis and Inhibits Tumor Cell Proliferation in Mice

**FIGURE 6:**
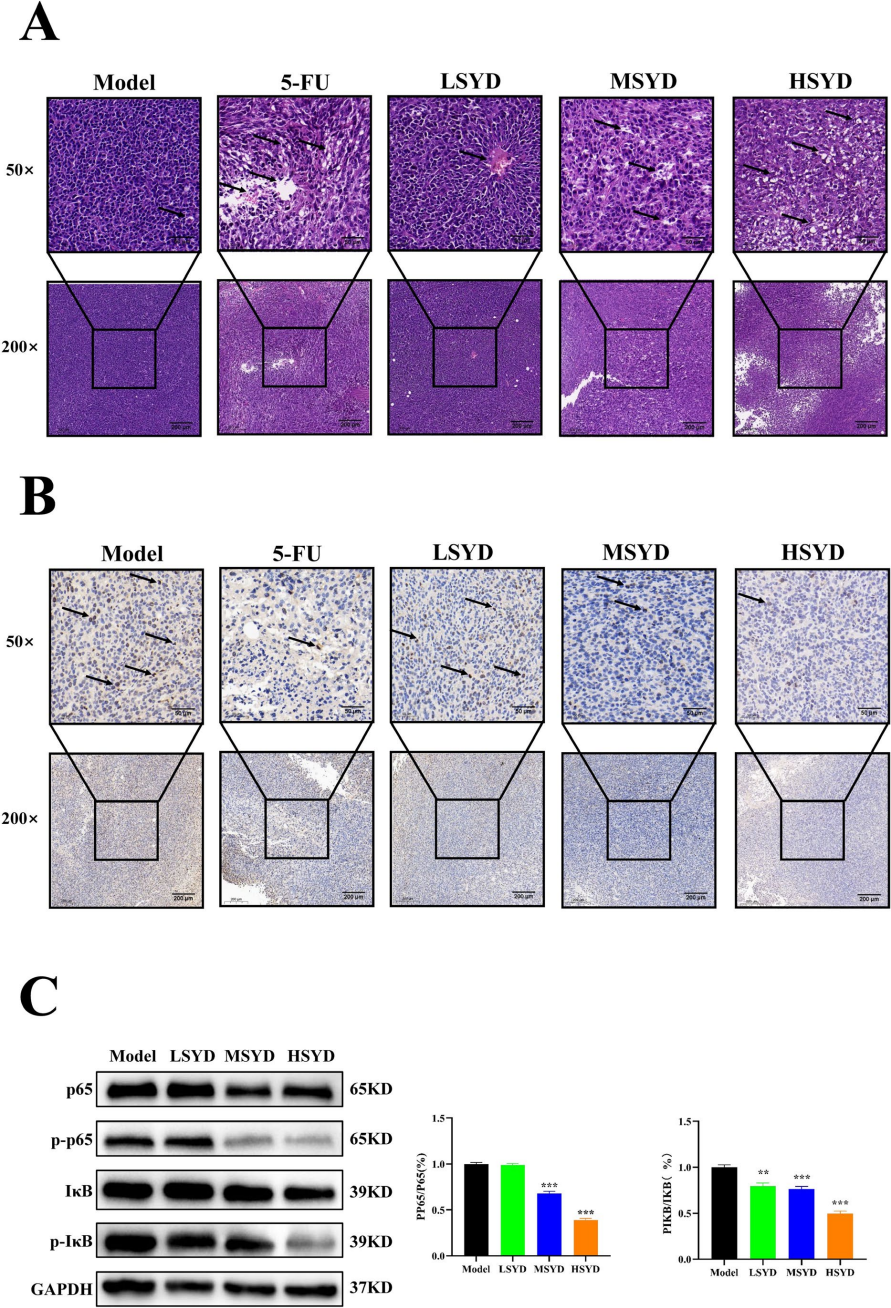
HE staining of the tumors, immunohistochemical analysis of Ki67, and Western blotting. The magnification was 200 × and 50 ×. (A) HE staining of the tumors, the arrow marking the area of tumor necrosis. (B) Immunohistochemical analysis of Ki67, the arrow marking the area of tumor cell proliferation. (C) The protein expression levels of the IL‐17/NF‐κB pathway. *p < 0.05, **p < 0.01, ***p < 0.001 vs. control group.

In the Model and LSYD groups, cells exhibited tight and irregular organization, with large and highly pigmented nuclei, distinct nuclear membranes and nucleoli, scant cytoplasm, abundant tumor cells, and minimal necrotic areas. Varying degrees of inhibitory effects were observed in the 5‐FU, MSYD, and HSYD groups, characterized by sparsely arranged tumor cells, decreased volume, blurred nuclear membranes and nucleoli, and comparatively higher necrotic regions, as shown in Figure 6A. IHC staining results, depicted in Figure 6B, revealed a significant increase in the number of Ki67‐positive cells in the Model and LSYD groups, with a reduction observed to different extents in the 5‐FU, LSYD, MSYD, and HSYD groups. The outcome suggests a potential significant inhibition of CRC tumor cell proliferation.

### 3.5 SYD Attenuates Intestinal Inflammation via IL‐17/TNF/NF‐κB Signaling Pathway in Tumor‐Bearing Mice

In network pharmacology, the KEGG enrichment analysis revealed the IL‐17 and TNF with its downstream NF‐κB as the primary inflammatory signaling pathway. As demonstrated in Figure 6C, Western blot analysis revealed that all groups treated by SYD significantly reduced the expression of phosphorylated p65 and IκB in comparison to the Model group, resulting in a reduced intestinal inflammatory response. Through modulation of the IL‐17/TNF/NF‐κB signaling pathway, SYD exhibited the potential to slow the growth of CRC tumors.

### 3.6 SYD Was Able to Regulate the GM of Tumor‐Bearing Mice, Increase Beneficial Bacteria, and Promote the Stability of the Intestinal Microenvironment

**FIGURE 7:**
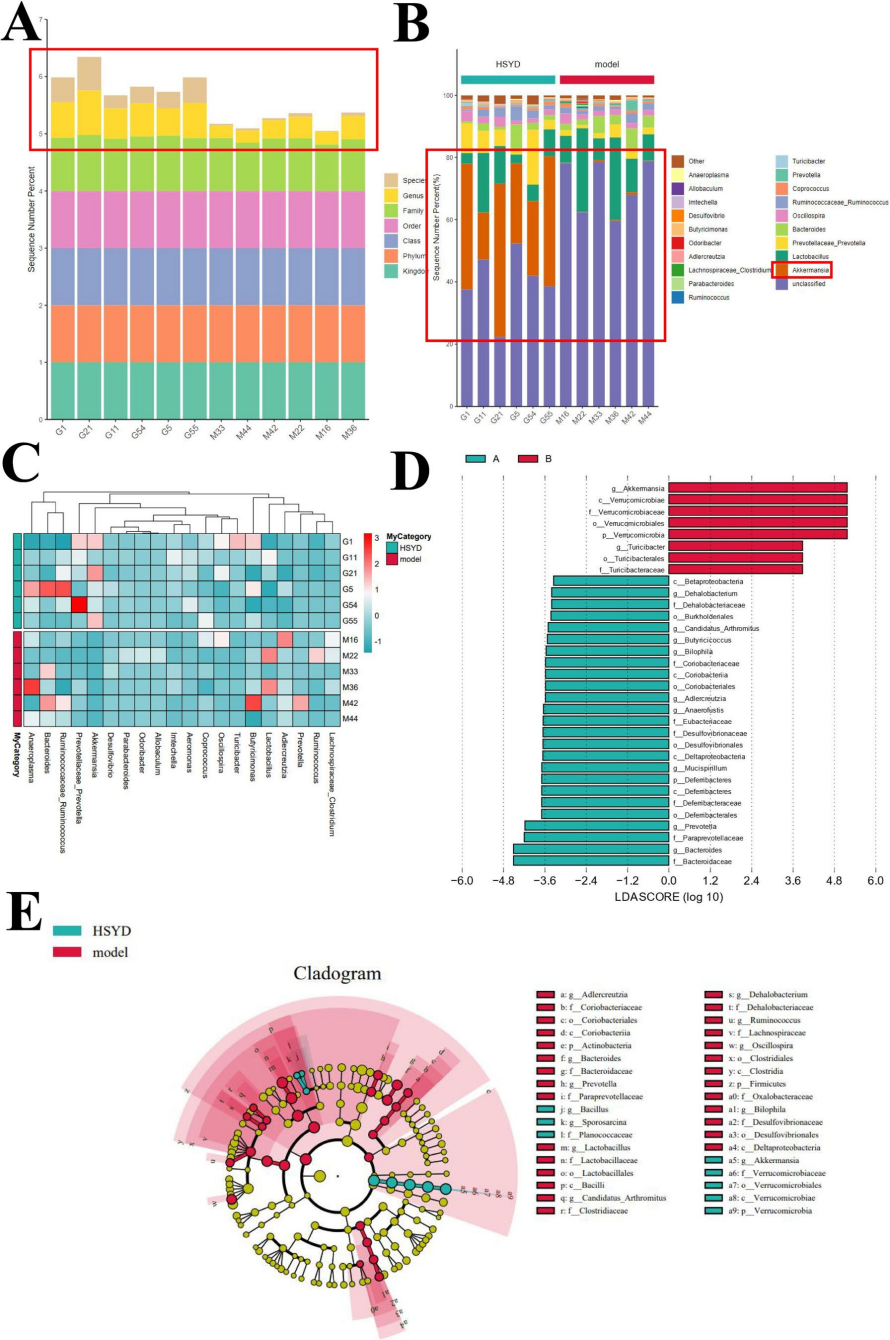
Diversity and structural analysis of the gut microbiome. (A) Biological distribution of different stages of tumorigenesis at the genus and species levels in the Model and HSYD groups. (B) Comparison of the abundance of the top 20 microbial species in the Model and HSYD groups. (C) Heat map of microbial richness clusters in the Model and HSYD groups. (D) Biology of discriminatory microorganisms with LDA scores > 3.5 or < −3.5 in the Model and HSYD group markers. (E) LEfSe branching plots indicating taxonomic unit enrichment in the microbiota of Model and HSYD groups.

**FIGURE 8:**
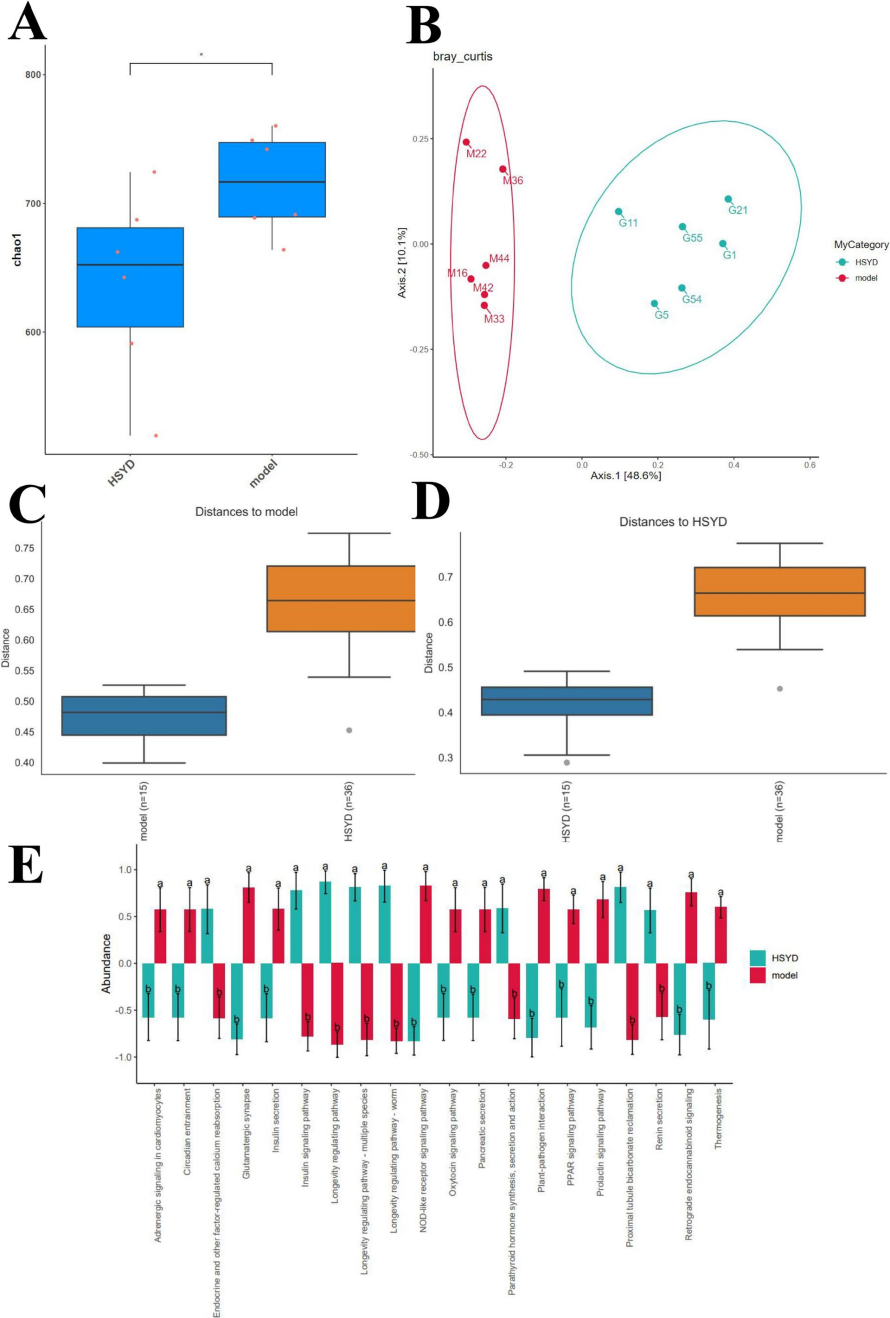
(A) Microbial community richness as shown by Chao1 index, indicating a fluctuating downward trend in the number of species during tumorigenesis. (B) PCoA based on the Bray–Curtis distance reveals that the microbiota structures at different stages of tumor development differ from each other in the Model and HSYD groups. (C, D) Microbial differences between the Model and HSYD groups. (E) Microbial abundance in the Model and HSYD groups at different pathways.

Numerous bacteria have been implicated in the onset and progression of illnesses in the human gut. The development and progression of CRC were directly correlated with the imbalance of gut microbes [30]. In line with this, the HSYD group exhibited significantly greater microbial diversity at the genus level than the Model group, according to the analysis of alterations in GM composition illustrated in Figures 7 and 8. Akkermansia levels were considerably increased in the HSYD group compared to the Model group, while the Model group displayed a higher overall abundance of bacterial communities than the HSYD group. The α diversity revealed that bacterial populations between the Model group and HSYD group differed statistically significantly (*p* < 0.05). The β diversity of microbiota showed a large difference in similarity between the above two groups of mice. The R value obtained from Anosim analysis was 0.972222, which indicated that the inter‐group difference exceeded the intra‐group difference and demonstrated statistical significance (*p* < 0.002). Overall, SYD stabilized the intestinal milieu, promoted GM alterations in tumor‐bearing mice, and decreased the onset and progression of CRC.

## 4 Discussion

Currently, CRC is the most prevalent malignancy in the digestive tract. Early‐stage CRC may manifest with altered defecation patterns, mucoid bloody stool, and rectal tenesmus when the integrity of the intestinal tract is compromised. Positive fecal occult blood test results, along with elevated levels of tumor‐related markers (such as CEA and CA199) are indicative features [31]. Imaging, endoscopy, and pathology techniques are essential for the accurate diagnosis of CRC [32]. The prevailing view in modern medicine suggests that most cancers evolve from chronic inflammatory conditions, with the molecular mechanisms of inflammation‐induced tumorigenesis regarded as crucial in blocking the development of cancer. In contrast, Traditional Chinese medicine attributes disease malignancy to deficiencies in positive energy and imbalances in qi, blood, yin, and yang during disease progression. Referring to the traditional Chinese medicine theories, the classical formula SYD, known for its heat‐clearing, dampness‐drying, and qi‐blood regulating properties, has been shown to not only slow down inflammation‐driven cancer transformation but also restore the balance of qi, blood, yin, and yang, thereby enhancing positive qi. SYD has been found to reduce key inflammatory markers such as IL‐1, IL‐6, and TNF‐α, resulting in decreased tumor‐associated macrophage (TAM) infiltration and NF‐κB activation, thereby inhibiting Snail‐induced epithelial–mesenchymal transition (EMT) and subsequently CRC progression [33]. Additionally, SYD exerts control over the IL‐17/TNF/NF‐κB signaling pathway, leading to the prevention of tumor cell differentiation, reduced cell proliferation, and alleviated mouse intestinal inflammation. Furthermore, the therapeutic effect of SYD was concentration‐dependent. Although current research has shown promising results in optimizing the approach to CRC treatment and inhibiting inflammatory responses with SYD, further pharmacological studies are needed to elucidate its interaction with the microbiota and clarify its underlying mechanisms through approaches such as GM analysis.

According to research, the occurrence and progression of CRC arise from a complex interplay between environmental factors, dietary habits, genetic predispositions, inflammatory bowel disease (IBD), and GM dynamics [34]. Diet and lifestyle are two broad elements that might alter the composition and quantity of GM [35]. CRC develops due to an imbalance in the GM and colony structure, leading to cellular DNA damage and activation of associated carcinogenic pathways [36]. Numerous studies have demonstrated the importance of GM regulation in the management of CRC [37, 38, 39].

An early pharmacological research has demonstrated that SYD effectively alleviates chemical colitis by triggering GPX4, preventing iron ptosis in epithelial cells, and further restoring barrier function, and furthermore determined that Wogonoside, Wogonin, Palmatine, Paeoniflorin, and Liquirin are the main medicinal substances responsible for SYD's anti‐colitis actions [40]. Through network pharmacology screening and LC–MS analysis, 13 potential active compounds of SYD, 8 Hub genes, and 3 important pathways were discovered in the present study. Among these, extracts containing flavonoids such as Wogonin have shown the potential to ameliorate gut inflammation and cancer. To improve the outcomes of chronic colitis, Wogonin reclaimed intestinal barrier dysfunction via restoring the lymphoid cells balance of ILC3/ILC1 by binding to aryl hydrocarbon receptor (AhR) directly and activating the AhR pathway indirectly by altering the tryptophan metabolisms of GM [41]. Another research revealed that Wogonin inhibited the phosphorylation of AKT1 in CRC cells, thereby suppressing cellular proliferation and invasiveness, triggering apoptosis in CRC cells, and delaying the EMT, a crucial physiological process that governed the initial stages of CRC cells invasion and migration [42]. Daidzein intake has been associated with the production of intestinal microbes that exhibit inhibitory effects on tumor growth and progression [43, 44]. Genistein has been shown to augment the presence of lactobacilli in the feces while concurrently reducing Salmonella levels, thereby alleviating colitis and inhibiting loss of cup cells due to Salmonella infection [45]. Trans‐cinnamic acid (tCA) was indicated that potentially inhibited histone deacetylases (HDACs), whose overexpression is associated with tumor initiation and progression, to interfere in histone acetylation then leading to colon carcinoma cell cytotoxicity in nude mice [46]. Epitestosterone, 2‐(5‐mercapto‐4‐methyl‐4H‐1,2,4‐triazol‐3‐yl)acetonitrile, propylparaben, leucylproline, valylproline, 1‐methyladenosine, 3‐[4‐methyl‐1‐(2‐methylpropanoyl)‐3‐oxocyclohexyl]butanoic acid, DL‐α methoxyphenylacetic acid, and 1‐phenyl‐3‐methyl‐5‐pyrazolone have not been reported with GM‐related content names.

The total of eight hub genes, IL6, TNF, JUN, CASP3, MMP9, CXCL8, PTGS2, and PPARG, are predominantly associated with inflammatory responses, playing pivotal roles in both inflammatory processes and immune regulation [47, 48, 49, 50, 51]. It has been shown that the development of CRC is closely related to the expression of inflammatory factors [52]. Reducing the expression of inflammatory factors inhibits the phosphorylation of IκK and IκB, as well as NF‐κB proteins. This suppression regulates the NF‐κB signaling pathway, thereby inhibiting CRC proliferation and promoting apoptosis and autophagy [53]. It has also been shown that these inflammatory factors are associated with GM disorders and intestinal immunity. Studies found that altered flora in patients with neonatal necrotizing small bowel colitis led to significant intestinal inflammation, resulting in increased expression of IL‐1, IL‐2, IL‐4, IL‐6, IL‐8, IL‐10, TNF‐α, IFN‐γ, and IL‐17 in samples [54]. The differentiation of cytokines associated with the IL‐17 signaling pathway has been linked to GM colonization and may contribute to intestinal immune homeostasis [55]. It has been suggested that Lipid and atherosclerosis are associated with microbial metabolites SCFAs, especially butyrate, which may have implications for various inflammatory mechanisms [56]. It has been shown that changes in gut microbes are associated with the NOD/RIP2/NF‐κB signaling pathway at different levels of intestinal injury [57].

In addition to the IL‐17 signaling pathway, our enrichment analysis identified several other pathways mechanistically linked to CRC progression. The TNF signaling pathway induces apoptosis in colon cancer cells, such as SW‐480, thereby inhibiting their proliferation [58]. This suggests a protective role against CRC by limiting tumor cell growth. Similarly, the Toll‐like receptor 4 (TLR4) signaling pathway contributes to CRC by promoting macrophage activation and infiltration into the colonic mucosa, as well as increasing proinflammatory cytokine expression, fostering an inflammatory microenvironment conducive to tumor development [59]. Inhibiting this pathway can reverse these effects, underscoring its role in CRC pathogenesis. Furthermore, the C‐type lectin receptor signaling pathway is essential for maintaining immune homeostasis in the gut [60]. Dysregulation of this pathway can exacerbate colitis and promote colon tumor formation, while its modulation may inhibit CRC progression. Lastly, the AGE‐RAGE signaling pathway, activated by oxidative stress and dietary factors, plays a crucial role in CRC by driving inflammation and cellular dysfunction [61]. Targeting this pathway could offer therapeutic benefits for CRC treatment by mitigating these tumor‐promoting effects. Apart from the aforementioned pathways, the other top 20 results in the enrichment analysis (such as Salmonella infection, pertussis, non‐alcoholic fatty liver disease, measles, hepatitis B, Amoebiasis, alcoholic liver disease, Leishmaniasis, Legionellosis) are either unrelated to CRC or lack supporting literature evidence for their association with CRC.

Previous studies have shown that SYD significantly increased the survival rate of the mice, ameliorated the general well‐being of the mice, reduced the incidence and multiplicity of colonic neoplasms, and inhibited EMT [62]. Similarly, in the present study, few adverse effects were observed in the experimental animals, and among the SYD gavaged groups, the middle‐dose group and high‐dose group apparently exhibited fewer and smaller tumors than did the Model group in gross evaluation. According to histopathological evaluation, the HE staining of the tumor tissue shows a large area of necrosis, and the IHC staining shows a low expression level of Ki‐67, an antigen related to cell proliferation. These results indicated that SYD could effectively suppress colitis‐associated tumor growth by inhibiting proliferation and promoting apoptosis. Other studies have mentioned that the CRC subcutaneous graft tumor mice model established using CT26 cells were prone to colorectal cancer liver metastasis (CRLM) and liver injury, leading to hepatomegaly [63, 64]. Notably, in the present study, it was observed that the liver weight of mice in the model group was significantly higher than that of mice in the 5‐FU treatment group and the low‐, middle‐, and high‐dose SYD treatment groups, suggesting that 5‐FU and SYD might reduce liver damage and inhibit CRLM. Reports suggested that 5‐FU reduced the detectable tumor numbers of liver metastasis, delaying the progression of CRLM [65]. However, our study lacked observational evidence of pathological changes in the liver and detection of metabolic changes to support the above speculations.

According to the analysis of microbial diversity, SYD has been found to alter microbial diversity and species abundance in tumor‐bearing mice, potentially influencing microbiological outcomes. Notably, SYD significantly increased the abundance of Akkermansia, a specific GM associated with CRC or animal models infected with particular pathogens, characterized as Gram‐negative anaerobic bacteria [66]. Akkermansia exhibits a protective effect on reducing mucosal inflammation in DSS‐induced colitis model mice, primarily through preserving the integrity of the intestinal epithelial barrier, mitigating microbial host interactions associated with inflammatory cytokine levels, and promoting a healthy microbiota composition [67]. Akkermansia decreases macrophage activity and augments the efficacy of PD‐1 blockade on tumor growth [68]. It also exhibits potential in mitigating CRC onset and progression through the reduction of the Bacillota/Bacteroidota ratio [69]. Akkermansia produces the acetyltransferase Amuc_2172, which reprograms the tumor microenvironment by inducing the secretion of heat‐shock protein 70 (HSP70) and promoting the immune response mediated by CD8+ cytotoxic T lymphocytes (CTLs) during tumorigenesis [70]. According to emerging studies, Akkermansia colonization bolsters NLRP6 function, fosters autophagy, ensures consistent antimicrobial peptide secretion by Paneth cells, augments the expression of tight junction proteins, negatively moderates the NF‐κB signaling pathway, and curtails the expression of inflammatory cytokines, thereby fortifying the mucosal barrier's defensive mechanisms [71]. This bacterium enhances mucin production and diminishes inflammatory cytokines by impeding NF‐κB signaling [72]. Clinical data reveal that Akkermansia is markedly less prevalent in the feces of colitis patients [73], alleviating DSS‐induced colitis by GM modulation and NF‐κB pathway regulation [74]. Such findings imply that Akkermansia's presence is crucial for NF‐κB pathway activation. Nevertheless, numerous studies have identified a higher abundance of Akkermansia in CRC patients and mice compared to healthy counterparts [75, 76], necessitating further exploration into the association between Akkermansia and CRC. Despite this paradox, emerging evidence supports a tumor‐suppressive role for Akkermansia. The abundance of *A. muciniphila* is significantly reduced in patients with CRC, and supplementing *A. muciniphila* can inhibit the occurrence of colonic tumors in ApcMin/+ mice and suppress the growth of implanted HCT116 or CT26 tumors in nude mice [77]. An increase in *A. muciniphila* has also been observed to inhibit the proliferation of HCT‐116 and HT‐29 tumors in normal Balb/c mice [78]. Mechanistically, Akkermansia exerts its anti‐tumor effects through dual pathways. First, its outer membrane protein‐derived Amuc activates the Toll‐like receptor 2 (TLR2) signaling pathway, thereby mediating anti‐tumor immune responses [79]. Second, supplementation with *Akkermansia muciniphila* ( *A. muciniphila* ) specifically inhibits the tryptophan‐mediated aryl hydrocarbon receptor/β‐catenin (AhR/β‐catenin) signaling pathway, conferring protection against the development and progression of CRC in murine models [80].

Currently, the therapeutic concept of microbiota intervention to alleviate CRC in patients with CRC combined with GM dysbiosis is highly promising. A multifunctional oral nanomedicine (P3C‐Asp), constructed by covalently linking a reactive oxygen species (ROS)‐sensitive aspirin prodrug (PBAsp) with dextran, significantly increased the relative abundance of GM associated with enhanced anti‐tumor immune responses in BALB/c mice. Specifically, the abundance of Lactobacillus and Akkermansia was elevated by 6.7‐fold and 103‐fold, respectively, while the abundance of pathogenic bacteria, such as Bacteroides, was reduced [81]. Immunotherapy combined with chemotherapy or probiotic regimens can significantly improve the prognosis of patients with mCRC by restoring GM balance and enhancing intestinal function, while reducing the incidence of adverse effects [82]. Their study demonstrated the ability of SYD to reverse gut dysbiosis in CRC, and it is foreseeable that SYD could be combined with other therapies (e.g., probiotic therapy) as an important adjunct to cancer treatment strategies.

**FIGURE 9:**
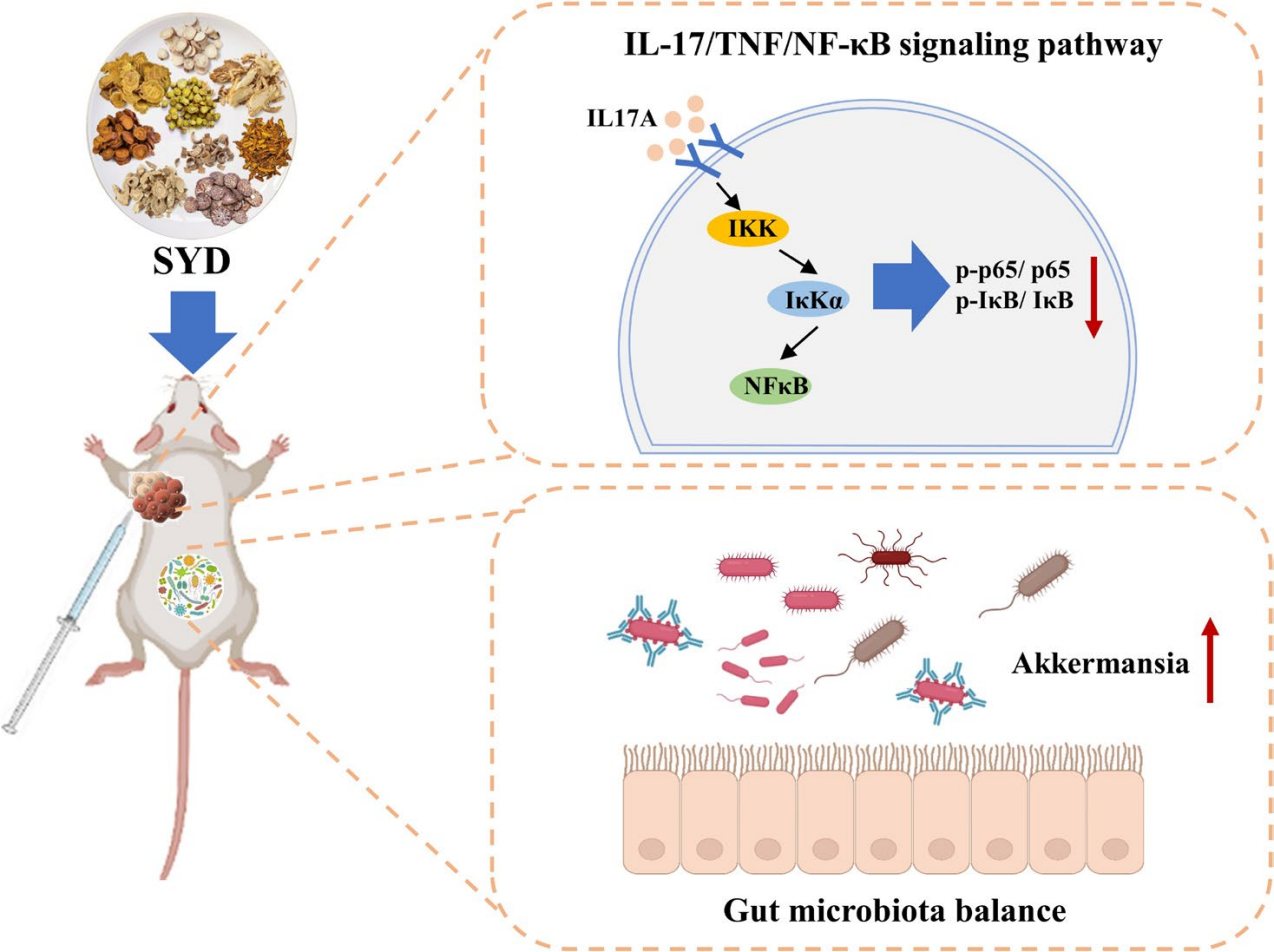
The inhibitory mechanism of SYD on tumor cell proliferation in mice.

In this study, we utilized a combination of network pharmacology, molecular docking techniques, and in vivo experiments to demonstrate that SYD effectively improved the intestinal inflammatory environment, inhibited CRC cell proliferation, and promoted its apoptosis by suppressing the phosphorylation of the IL‐17/TNF/NF‐κB signaling pathway. Furthermore, 16 s rRNA sequencing analysis disclosed that SYD influenced the activity of CRC cells by significantly increasing the abundance of Akkermansia, a type of bacterium that mitigated intestinal mucosal inflammation, protected the intestinal epithelium, and mediated specific immune responses to achieve reprogramming of the tumor microenvironment. Notably, SYD might reduce liver damage and delay liver metastasis of CRC through certain pathways, although further exploration at the microscopic level is needed to elucidate its mechanisms. In summary, our study provided evidence for the ability of SYD to regulate GM to inhibit the growth and metastasis of CRC cells (Figure 9). As expected in this study, the traditional Chinese medicine formula SYD could serve as a promising novel adjuvant therapy for CRC. However, the multi‐component, multi‐target, and multi‐pathway anti‐tumor mechanisms of traditional Chinese medicine should not be ignored. Therefore, further exploration of the mechanisms by which SYD participates in other pathways against CRC, conducting mechanism studies of SYD on animal models of colorectal primary tumors, and large‐scale real‐world clinical studies of SYD are of great significance.

## 5 Conclusion

In summary, our experiments revealed that by regulating the composition of GM, such as Akkermansia, SYD might interact with the IL‐17/TNF/NF‐κB signaling axis, enhancing intestinal immunity, metabolic pathways, and inflammatory responses to influence the development and progression of CRC. Additionally, SYD has demonstrated the potential to alleviate liver damage and inhibit tumor liver metastasis, but its mechanism awaits further exploration. The microbiota–immune interaction highlights SYD as a promising complementary approach to standard therapies, especially for CRC patients experiencing gut dysbiosis.

## Author Contributions

**Yaojun Rong:** conceptualization (equal), formal analysis (lead), investigation (lead), visualization (lead), writing – original draft (equal). **Guiyu Zhang:** conceptualization (equal), data curation (lead), methodology (lead), resources (lead), writing – original draft (equal). **Wenhao Ye:** investigation (equal), visualization (equal), writing – review and editing (equal). **Linhua Qi:** investigation (equal), writing – review and editing (equal). **Xiaojiang Hao:** investigation (equal), writing – review and editing (equal). **Xiaolin Li:** investigation (equal), writing – review and editing (equal). **Wuhong Zhang:** investigation (equal), writing – review and editing (equal). **Yangfa Chao:** funding acquisition (equal), supervision (lead), writing – review and editing (equal). **Shaodong Gu:** funding acquisition (equal), project administration (lead), writing – review and editing (equal).

## Disclosure

Publisher's note: All claims expressed in this article are solely those of the authors and do not necessarily represent those of their affiliated organizations, or those of the publisher, the editors, and the reviewers. Any product that may be evaluated in this article, or claim that may be made by its manufacturer, is not guaranteed or endorsed by the publisher.

## Ethics Statement

Ethical approval was granted by Shenzhen Top Biotech Co. Ltd., Institutional Animal Care and Use Committee, IACUC (No. TOP‐IACUC‐2021‐0125, No. TOP‐IACUC‐2021‐0126).

## Conflicts of Interest

The authors declare no conflicts of interest.

## Supporting information


Data S1.

## Data Availability

The data for this article will be made available on request.
