# Supplementary material for: Uncovering the Effects and Molecular Mechanisms of Shaoyao Decoction Against Colorectal Cancer Using Network Pharmacology Analysis Coupled With Experimental Validation and Gut Microbiota Analysis
Source: Cancer Med. 2025 Mar 22;14(6):e70813. doi: 10.1002/cam4.70813 (PMC11928771; doi:10.1002/cam4.70813)
Supplement: Supplementary file 1 — Data S1. [file CAM4-14-e70813-s001.docx]

**Supplementary Material S1**

**Antibodies and Main Reagents in the Research**

**Beyotime** (Shanghai, China) provided 5-fluoropyrimidine (5-FU, #ST1060-1g) and protease and phosphatase inhibitor cocktail 50X (#P1045).

**Biosharp** (Hefei, China) produced 4% paraformaldehyde (#BL539A).

**Dean Gene** (Guangzhou, China) offered ordinary maintenance feed(#1110-1) and corn cob padding (#1110).

**Thermo Fisher Scientific** (Waltham, MA, USA) distributed RIPA lysis and extraction buffer (#89901), BCA Protein Assay Kit (#23225).

**Cell Signaling Technology** (Danvers, MA, USA) provided phospho-IκBα (Ser32) (14D4) rabbit monoclonal antibody (#2859S), IκBα (44D4) rabbit monoclonal antibody (#4812S), phospho-NF-κB p65 (Ser536) (93H1) rabbit monoclonal antibody (#3033S), and NF-κB p65 (D14E12) XP rabbit monoclonal antibody (#8242S).

**Sanying** (Wuhan, China) supplied GAPDH rabbit polyclonal antibody (#10494-1-AP).

**Bioworld** (Nanjing, China) provided goat anti-rabbit IgG (H+L) labeled by Horseradish Peroxidase (HRP) (#BS13278).

**Sinopharm** (Beijing, China) provided absolute ethanol (#100092683), xylene (#10023418), N-butanol (#100052190), neutral gums (#10004160), hydrochloric acid (#10011028),

**Anjie Hi-Tech** (Shandong, China) offered 3% hydrogen peroxide disinfectant (#D.40036)

**Servicebio** (Wuhan, China) offered Environmentally friendly dewaxing solution (#G1128), Universal tissue fixative (#G1101), Hematoxylin-eosin (H&E) HD constant staining kit (#G1076), environmentally friendly dewaxing transparent liquid (#G1128), 20× citric acid antigen retrieval solution (pH 6.0) (#G1202), 20× Tris-EDTA antigen retrieval solution (pH 9.0) (#G1203), 20× Tris-EDTA antigen retrieval solution (pH 8.0) (#G1206), PBS buffer (#G0002), universal tissue fixative solution (Neutral) (#G1101), bovine serum albumin (BSA) (#GC305010), normal rabbit serum (concentrated) (#G1209), Hematoxylin staining solution (#G1004), Hematoxylin differentiation solution (#G1039), Hematoxylin blue solution (#G1040), Ultra-clean and quick-drying mounting gel (#G1404-100mL), Histochemistry kit DAB chromogen (#G1212) and anti-Ki67 rabbit pAb (#GB111141-100).

**Macklin** (Shanghai, China) offered CTAB (#H811115).

**Tsingke** (Beijing, China) provided 1% agarose gel electrophoresis (#TSJ001)

**Illumina** (San Diego, USA) offered TRUSEQ®DNA PCR-Free Sample Preparation Kit (#20015963)

**BIORAD** (Hercules, CA, USA) offered 5× Protein Loading Buffer (#1610374) and Pre-stained Marker Protein (1610394).

**Merck Millipore** (Billerica, MA, USA) provided PVDF 0.22um membrane (#ISEQ00010) and ECL Development and Fixing Reagent (#WBKLS0500)

**BD** (Franklin Lakes, NJ, USA) provided Skimmed Milk Powder (#232100)
